# Measuring changes in adult health and well-being during the COVID-19 pandemic and their relationship with adverse childhood experiences and current social assets: a cross-sectional survey

**DOI:** 10.1186/s12889-023-16549-z

**Published:** 2023-08-24

**Authors:** Mark A. Bellis, Karen Hughes, Kat Ford, Helen Lowey

**Affiliations:** 1 Faculty of Health, Liverpool John Moores University, Liverpool, L2 2ER UK; 2 World Health Organization Collaborating Centre on Investment for Health and Well-Being, Policy and International Health, Public Health Wales, Wrexham, LL13 7YP UK; 3 Public Health Collaborating Unit, School of Medical and Health Sciences, College of Human Sciences, Bangor University, Wrexham, LL13 7YP UK; 4 Helen Lowey Consultancy Ltd, Lathom, L40 4BQ UK

**Keywords:** Adverse childhood experiences, COVID-19, Resilience, Mental health, Physical health, Sleep

## Abstract

**Background:**

Adverse childhood experiences (ACEs) can impact mental and physical health, leaving people with less resilience to health challenges across the life-course. This study examines whether individuals’ levels and changes in levels of mental health, physical health and sleep quality reported across the first year of the COVID-19 pandemic are associated with ACEs and moderated by social assets such as having trusted family and friends.

**Methods:**

A cross-sectional household telephone survey in England (a North West local authority) and Wales (nationally) using landline and mobile numbers stratified by health areas, deprivation quintile and age group and supplemented by an online survey. Data were collected from 4,673 English and Welsh residents aged ≥ 18 years during national COVID-19 restrictions (December 2020 to March 2021). Measures included nine types of ACE; self-reported mental health, physical health and sleep quality at time of survey (in pandemic) and one-year earlier (pre-pandemic); numbers of trusted family members and friends, knowledge of community help; and COVID-19 infection.

**Results:**

ACEs were strongly related to moving into poorer mental health, physical health, and sleep categories during the pandemic, with likelihoods more than doubling in those with ≥ 4 ACEs (vs. 0). ACEs were also associated with increased likelihood of moving out of poorer health and sleep categories although this was for a much smaller proportion of individuals. Individuals with more trusted family members were less likely to move into poorer health categories regardless of ACE counts.

**Conclusions:**

ACEs are experienced by large proportions of populations and are associated with poorer health even in non-pandemic situations. However, they also appear associated with greater vulnerability to developing poorer health and well-being in pandemic situations. There is a minority of those with ACEs who may have benefited from the changes in lifestyles associated with pandemic restrictions. Connectedness especially with family, appears an important factor in maintaining health during pandemic restrictions.

**Supplementary Information:**

The online version contains supplementary material available at 10.1186/s12889-023-16549-z.

## Background

Increasing evidence links adverse childhood experiences (ACEs) to poorer health, social and economic outcomes across the life-course [1–3]. ACEs include child maltreatment (physical, sexual and emotional) and other chronic stressors such as exposure to domestic violence and substance use in the home environment [4]. Around half of adults in Europe and North America have experienced at least one ACE and many have suffered multiple ACEs [5] (e.g. 8–13% with ≥ 4 ACEs in UK [6]). Exposure to ACEs is strongly associated with increased risks of adopting health-harming and anti-social behaviour (e.g. substance misuse, violence), developing poor mental health and displaying lower resilience to sources of stress [2, 7]. Longer term, a history of ACEs increases risks of cancer, cardiovascular disease and other non-communicable diseases throughout adulthood [2, 3]. Biomedical research continues to identify changes in neurological, hormonal, immunological and epigenetic markers consistent with harmful impacts of ACEs on health and well-being [8–10]. However, studies also identify that a range of other experiences throughout life can moderate or even negate some of the increased health and social risks associated with ACEs [11, 12]. Thus, individuals’ ability to cope with acute and longer-term challenges to health and well-being may be affected by factors such as support from social assets including family, friends and community services, with greater social assets potentially reducing risks of poorer health [13].

For whole populations, the COVID-19 pandemic introduced radical change to people’s lives. Individuals were exposed to a protracted threat of infection from a potentially fatal virus. Workplaces and educational establishments were closed, threatening livelihoods, career plans and future economic prospects. Individuals had behaviours such as mask wearing imposed on them and were frequently banned from physically meeting friends and family, leaving only virtual communication as an option for some and resulting in social isolation for others. Moreover, professional and voluntary services for those with health and social support needs were reduced and sometimes removed [14, 15]. Whilst studies have examined how such changes have impacted people’s physical and mental health [16, 17], and the protective effects of social assets [18], few have considered whether such impacts may be exacerbated or even moderated by a history of ACEs. Poorer physical and mental health, potentially resulting from ACE exposure, may leave people with increased vulnerability to further harm associated with pandemic-related worries and restrictions. However, some studies link ACEs to social anxiety and a preference for greater interpersonal distance [19, 20]. Thus, pandemic conditions for some may even create a respite from unwanted professional and social interactions and attention.

Understanding what represents risk or protective factors for physical and mental health during pandemics or other crises is important for all individuals. However, those with ACEs and consequently who have already experienced factors linked with developing poorer mental and physical health [2], may be even more vulnerable to stressors arising from the pandemic. Therefore, it is important to identify specifically how periods of pandemic restrictions may affect their health and well-being. Such information can help inform responses to future pandemic threats as well as identifying populations that may still require additional support as a result of experiencing the COVID-19 pandemic. Therefore, here we measure three aspects of self-reported well-being – mental health, physical health and quality of sleep – approximately 12 months into the pandemic in England and Wales and retrospectively for a year prior (i.e. pre-pandemic). We test the hypotheses that a history of ACEs is associated with individuals reporting poorer scores for each well-being measure both prior to the pandemic and one year on. In additional, we test the hypothesis that ACEs are also associated with changes in health and well-being measures during the pandemic, resulting in greater proportions of those with ACEs moving into or out of poorer outcome levels across the study period.

## Methods

### Data collection

A telephone survey was conducted between December 2020 and March 2021 in Wales (national) and England (Bolton Local Authority) with residents aged ≥ 18 years. Data collection coincided with a period of national COVID-19 restrictions in both Wales and England, which limited social contact through social distancing, bans on household mixing, and closure of hospitality and some non-essential retail, and required the use of face coverings in indoor public places. A target sample of 2,000 in each study location was set to capture adequate individuals across ACE categories, with a minimum of 200 respondents with high (≥ 4) ACEs in each locality [6].

Following initial testing and refinement of the survey tool by the research team, a professional market research company (MRC) was appointed to undertake further piloting (*n* = 61) and data collection using a random stratified sampling approach. Telephone contacts (landline and mobile) were obtained from a commercial sample provider and, in Wales, stratified by region (Welsh Health Board area). Contacts were then stratified by residential deprivation based on rankings in the English and Welsh Indexes of Multiple Deprivation [IMD] [21, 22] and age group. The IMD is a routinely used measure of socioeconomic status for residents in a locality, although variations exist in the methodologies for England and Wales. In order to address difficulty accessing younger age groups using telephone sampling, an online version of the survey was also developed. This was disseminated through an online panel (individuals paid to take part in online research) accessed through a commercial provider and also promoted in England through colleges and other local services. Although aimed at engaging younger individuals, the online panel was extended to all adult age groups proportionate to population demographics.

Study inclusion criteria were aged ≥ 18 years, resident in study area and cognitively able to participate. A description of the study, including its purpose and the types of subjects covered by the questionnaire, was verbally provided to potential participants on contact by telephone. Potential participants were also informed about the survey’s voluntary, anonymous, and confidential nature. Those completing the survey online were provided with this information electronically. Potential participants were informed that they did not have to answer all questions and could withdraw at any point. Informed consent was recorded as part of the survey using opt-in consent (verbally or electronically depending on method). Following survey completion, contact details for the research team and appropriate support services were provided (telephone participants were provided with a web-link). All study materials were available in English, and in Wales, Welsh language. Telephone calls were made between 9am-9pm on weekdays and 10am-4pm on weekends. On average, the survey took 20 min to complete.

Telephone contact was made with 12,536 individuals, of whom 230 (1.8%) did not meet the inclusion criteria and 7,964 (63.5%) declined. Of those who agreed to participate (*n* = 4,342), 358 did not meet the age quota in their area and 3,984 completed the questionnaire. Thus, the telephone participation rate was 33.3% (3,984/11,948) of eligible individuals who met the quota sampling, or 32.4% (3,984/12,306) of all eligible telephone participants. We were unable to calculate a participation rate for the online sample. However, 887 participants completed the survey online (237 England; 650 Wales), leading to a total combined sample of 4,871. We used a complete case analysis approach to missing data with individuals who had not completed all questions relevant to this study being removed (4.1% of the total combined sample). Data were also restricted to cases identifying as males and females, due to very low numbers (*n* = 2) reporting other genders, leaving a final sample for analysis of 4,673.

### Study questionnaire

All measures were self-reported. The full questions and response options used for study outcomes are provided in Additional file 1: Table A1. An adapted version of the Centers for Disease Control and Prevention short ACE tool [23] was used to measure exposure to nine ACE types (before age 18 years; physical, verbal and sexual abuse; parental separation; exposure to domestic violence; and living with a household member with mental illness, alcohol abuse, drug abuse or who was incarcerated). In line with international literature, individuals were categorised by ACE count (0 ACEs, 1 ACE, 2–3 ACEs, ≥ 4 ACEs; [2]). In order to provide consistency for respondents, individuals were asked to rate each outcome (mental health, physical health and sleep) on a 0–10 scale (mental health: 0 extremely poor to 10 extremely good; physical health: 0 not at all healthy to 10 completely healthy; and sleep: 0 not at all well to 10 extremely well). 0–10 scales are commonly used in surveys to rate and compare individuals’ health and well-being [24, 25]. Wording and categorisations were developed specifically for measuring these two COVID-related time periods in a consistent and succinct manner which also minimised survey length and so supported compliance in a pandemic setting. Participants answered the same questions for both the time periods; now (time of data collection; approximately one-year-on from the start of the pandemic) and a year ago (retrospectively pre-pandemic). Whilst this survey design, adapted for the pandemic period, allowed examination and comparison of multiple aspects of pre and in pandemic life, the limitations of these measures and retrospective data collection are addressed in the discussion.

To avoid any post hoc categorisation, for all measures, poorer outcomes were considered scores of ≤ 5. Thus, individuals were categorised as, for instance, *never* poorer mental health (≥ 6 pre-pandemic, ≥ 6 one year on); *always* poorer mental health (≤ 5 pre-pandemic, ≤ 5 one year on); *pre-COVID-only* poorer mental health (≤ 5 pre-pandemic, ≥ 6 one year on) and *one-year-on-only* poorer mental health (≥ 6 pre-pandemic, ≤ 5 one year on). The relative merits of this categorisation process are considered in the limitations.

Individuals were asked to report how many trusted family members and trusted friends (outside of their family) they currently had, with responses to both questions coded into none, 1–2, ≥ 2. Individuals also reported community support access with a positive answer indicating that they knew where to get help in their local community. Participants were asked if they thought they have had, or currently have, COVID-19; with those responding ‘yes’ categorised as *having had COVID-19*. Respondents’ age (five year groups), gender (male; female; other) and ethnicity (UK census categories) were also collected. For the purposes of analysis, age was categorised into ten-year groupings (18–29; 30–39; 40–49; 50–59; 60–69; 70 +) and due to low levels in individual non-white categories, ethnicity was re-categorised (white, other). Postcode of residence was captured by the MRC and converted into deprivation quintile using the respective IMD of the study area (1 = most deprived to 5 = least deprived). Whilst this study was structured to collect data from all adult age, sex and deprivation demographics it did not aim to provide a population representative sample for England and Wales. However, for context, demographic comparisons between the study sample and England and Wales adult population are provided in Additional File 1: Table A2.

### Statistical analysis

Statistical analyses used SPSS v27. Cross-tabulations and chi-square tests were used to examine initial relationships between outcome variables and ACEs and other participant characteristics (age, gender, ethnicity, deprivation, COVID-19 infection and social assets), study location (England, Wales) and method (telephone, online). Following study design, and to avoid any post hoc decisions regarding splitting data, all responses (telephone and online) were incorporated into individual models for each outcome of interest with a survey method variable included in the models. Independent associations between ACEs and outcome categories were measured using multinomial logistic regression (MLR), controlling for other participant demographics. For each outcome of interest, individuals not in the poorer outcome category at either time period (one year on, pre-COVID) were used as the reference category (i.e. *never* category). This allowed adjusted odds ratios for poorer well-being category membership one year on, pre-pandemic or at both time periods to be calculated for ACEs, social assets and other independent variables. The use of categorised variables avoided any assumption of linear or ordinal relationships between dependent and independent variables. In all MLRs, 0 categories are used as the reference for count variables (ACE count, numbers of trusted friends and family) and no is used as the reference for all yes/no variables (community help, had COVID). Based on the models fitted in Tables 1, 2 and 3 we generated socio-demographically adjusted percentages for category membership of *never*, *always*, *pre-COVID-only* and *one-year-on-only* for each outcome for specific socio-demographic groups. For the purposes of illustration figures use mid-deprivation (quintile 3), mid-age (40–49 years), white ethnicity and not having had COVID-19 for estimates presented in graphs. Graphs use stacked bars to show percentage membership of each poorer well-being category by time periods (*always*, *one-year-on-only* and *pre-COVID-only*) with movement out of poorer categories from pre-COVID to one-year-on represented below the x axis*.* Percentages are presented separately for each sex. Where effect sizes (Cohen’s d) are included, they are calculated from odds ratios with *d* values 0.2, 0.5, 0.8 considered small, medium and large effect sizes respectively [26].

**Table 1:** Percentage and adjusted odds ratios for self-rated poorer mental health according to the pandemic period

		Poorer mental health (%)					Adjusted odds ratios (95% confidence intervals)		
		Never	Always	Pre- COVID-only	One-year-on-only	P	Ref: *never* poorer mental health		
							Always	Pre-COVID-only	One-year-on-only
	n	3471	314	172	716				
	All	74.3	6.7	3.7	15.3				
ACE	0	80.9	4.9	2.4	11.8		Ref		
Count	1	76.4	5.7	3.3	14.6		0.94 (0.67–1.32)	1.32 (0.85–2.05)	1.11 (0.89–1.39)
	2–3	65.3	8.8	5.0	20.8		1.44 (1.04–2.01)	2.20 (1.42–3.38)	1.64 (1.30–2.06)
	≥ 4	51.6	14.3	8.9	25.2	***	2.36 (1.66–3.36)	4.36 (2.81–6.77)	2.11 (1.62–2.76)
Trusted	0	50.5	16.8	5.8	26.8		Ref		
Family	1	63.9	11.8	3.8	20.5		0.61 (0.35–1.05)	0.48 (0.21–1.10)	0.61 (0.39–0.94)
Members (n)	≥ 2	76.5	5.7	3.6	14.2	***	0.37 (0.23–0.60)	0.42 (0.21–0.83)	0.41 (0.28–0.60)
Trusted	0	64.0	14.3	2.8	19.0		Ref		
Friends (n)	1	62.1	13.2	3.7	21.0		0.90 (0.57–1.41)	1.23 (0.54–2.78)	1.05 (0.72–1.53)
	≥ 2	76.6	5.3	3.8	14.3	***	0.44 (0.31–0.64)	1.35 (0.69–2.63)	0.74 (0.55–1.00)
Community	No	68.6	10.5	4.0	16.9		Ref		
Help^a^	Yes	76.1	5.5	3.6	14.8	***	0.66 (0.50–0.86)	0.87 (0.60–1.25)	0.90 (0.74–1.10)
Had	No	75.8	6.5	3.7	14.1		Ref		
COVID-19	Yes	68.0	7.8	3.6	20.7	***	1.09 (0.81–1.48)	0.84 (0.56–1.26)	1.33 (1.09–1.63)
Deprivation	(least) 5	79.7	4.3	3.1	12.9		Ref		
Quintile	4	77.3	4.4	4.5	13.8		1.08 (0.68–1.71)	1.47 (0.89–2.41)	1.10 (0.83–1.46)
	3	76.9	6.9	3.2	13.0		1.42 (0.92–2.19)	0.95 (0.55–1.65)	0.91 (0.68–1.22)
	2	70.4	8.9	3.6	17.1		1.86 (1.22–2.83)	1.11 (0.65–1.89)	1.26 (0.95–1.66)
	(most) 1	68.6	8.7	3.9	18.8	***	1.78 (1.18–2.67)	1.15 (0.70–1.91)	1.32 (1.01–1.72)
Age	18–29	46.4	15.5	7.8	30.3		Ref		
(years)	30–39	59.5	11.6	6.4	22.4		0.59 (0.39–0.90)	0.65 (0.38–1.12)	0.57 (0.41–0.80)
	40–49	70.6	7.5	3.4	18.5		0.39 (0.26–0.59)	0.32 (0.18–0.56)	0.45 (0.33–0.61)
	50–59	73.3	6.8	3.9	16.0		0.33 (0.22–0.49)	0.37 (0.22–0.63)	0.39 (0.29–0.52)
	69–69	81.0	4.6	2.5	11.9		0.21 (0.13–0.33)	0.23 (0.13–0.41)	0.28 (0.20–0.38)
	70 +	89.3	2.4	1.9	6.4	***	0.11 (0.06–0.18)	0.19 (0.10–0.35)	0.15 (0.10–0.21)
Sex	Male	80.4	5.5	2.4	11.7		Ref		
	Female	70.6	7.4	4.4	17.5	***	1.51 (1.16–1.97)	1.99 (1.39–2.86)	1.71 (1.42–2.05)
Ethnicity	White	74.7	6.8	3.6	14.9		Ref		
	Other	66.7	5.9	5.2	22.2	**	0.50 (0.28–0.88)	1.31 (0.71–2.41)	1.08 (0.77–1.52)
Survey	Phone	76.7	5.4	3.5	14.4		Ref		
method	Online	61.0	13.9	4.8	20.3	***	1.90 (1.42–2.56)	1.28 (0.84–1.95)	1.29 (1.02–1.63)
Study	England	75.9	6.1	3.2	14.7		Ref		
location	Wales	73.2	7.1	4.0	15.7	ns	1.05 (0.81–1.38)	1.18 (0.83–1.68)	1.11 (0.92–1.34)

*ACE* Adverse childhood experience, *Ref* Reference category

*ns* Not significant

^a^Know where to get help in the community

^**^P < 0.01

^***^P < 0.001

**Table 2:** Percentages and adjusted odds ratios for self-rated poorer physical health according to the pandemic period

		Poorer physical health (%)					Adjusted odds ratios (95% confidence intervals)		
		Never	Always	Pre- COVID-only	One-year-on-only	P	Ref: *never* poorer physical health		
							Always	Pre-COVID-only	One-year-on-only
	n	3176	492	240	765				
	All	68.0	10.5	5.1	16.4				
ACE	0	74.8	8.8	4.0	12.4		Ref		
Count	1	67.9	9.2	5.3	17.5		1.02 (0.78–1.33)	1.28 (0.90–1.82)	1.39 (1.13–1.72)
	2–3	59.0	13.3	5.4	22.4		1.55 (1.18–2.03)	1.34 (0.91–1.97)	1.83 (1.47–2.29)
	≥ 4	48.9	17.3	10.0	23.9	***	2.08 (1.53–2.84)	2.54 (1.72–3.77)	2.01 (1.54–2.64)
Trusted	0	46.3	17.9	9.5	26.3		Ref		
Family	1	55.9	16.5	6.5	21.2		0.89 (0.54–1.46)	0.56 (0.29–1.09)	0.71 (0.46–1.10)
Members (n)	≥ 2	70.3	9.5	4.8	15.4	***	0.62 (0.40–0.97)	0.36 (0.21–0.64)	0.48 (0.32–0.70)
Trusted	0	56.3	20.5	5.3	18.0		Ref		
Friends (n)	1	52.8	18.6	5.4	23.2		0.97 (0.66–1.42)	1.00 (0.52–1.91)	1.34 (0.92–1.96)
	≥ 2	70.8	8.6	5.1	15.5	***	0.50 (0.37–0.68)	0.96 (0.58–1.60)	0.86 (0.63–1.16)
Community	No	62.9	15.2	4.9	17.0		Ref		
Help^a^	Yes	69.6	9.1	5.2	16.2	***	0.71 (0.57–0.89)	1.14 (0.82–1.59)	0.96 (0.79–1.17)
Had	No	69.5	10.7	4.8	15.0		Ref		
COVID-19	Yes	61.6	9.7	6.6	22.1	***	1.01 (0.78–1.32)	1.30 (0.94–1.79)	1.44 (1.18–1.76)
Deprivation	(least) 5	75.5	6.7	3.2	14.6		Ref		
Quintile	4	74.0	8.7	6.4	11.0		1.37 (0.96–1.96)	2.03 (1.28–3.22)	0.76 (0.57–1.01)
	3	70.1	9.3	5.6	14.9		1.45 (1.01–2.08)	1.71 (1.06–2.76)	0.99 (0.75–1.30)
	2	65.2	12.1	5.1	17.6		1.96 (1.39–2.78)	1.55 (0.95–2.53)	1.20 (0.92–1.56)
	(most) 1	57.8	14.7	5.4	22.1	***	2.96 (2.13–4.11)	1.74 (1.09–2.79)	1.67 (1.30–2.15)
Age	18–29	52.1	14.5	10.8	22.6		Ref		
(years)	30–39	58.5	11.0	8.8	21.6		0.73 (0.48–1.13)	0.76 (0.48–1.21)	0.88 (0.62–1.24)
	40–49	64.2	9.1	5.7	21.0		0.70 (0.46–1.04)	0.49 (0.31–0.79)	0.89 (0.65–1.23)
	50–59	66.4	10.6	4.6	18.4		0.80 (0.54–1.17)	0.40 (0.25–0.63)	0.81 (0.59–1.10)
	69–69	72.4	12.3	2.9	12.4		0.89 (0.60–1.30)	0.24 (0.14–0.41)	0.54 (0.39–0.76)
	70 +	78.5	8.4	3.3	9.8	***	0.59 (0.40–0.88)	0.29 (0.17–0.47)	0.43 (0.31–0.61)
Sex	Male	72.9	10.7	4.1	12.3		Ref		
	Female	65.0	10.4	5.8	18.8	***	1.15 (0.93–1.41)	1.52 (1.14–2.04)	1.68 (1.40–2.01)
Ethnicity	White	68.2	10.7	5.0	16.1		Ref		
	Other	64.1	7.8	7.0	21.1	*	0.58 (0.35–0.95)	1.00 (0.59–1.71)	0.97 (0.69–1.37)
Survey	Phone	70.4	8.8	4.8	15.9		Ref		
method	Online	54.5	19.9	6.9	18.8	***	2.15 (1.68–2.75)	1.44 (1.01–2.07)	1.26 (1.00–1.60)
Study	England	69.0	9.0	5.0	16.9		Ref		
location	Wales	67.3	11.5	5.2	16.0	ns	1.35 (1.09–1.69)	0.99 (0.74–1.33)	1.05 (0.88–1.26)

*ACE* Adverse childhood experience, *Ref* Reference category

*ns* Not significant

^a^Know where to get help in the community

^*^P < 0.05

^***^P < 0.001

**Table 3:** Percentages and adjusted odds ratios for self-rated poorer sleep according to the pandemic period

		Poorer sleep (%)					Adjusted odds ratios (95% confidence intervals)		
		Never	Always	Pre- COVID-only	One-year-on-only	P	Ref: *never* poorer sleep		
							Always	Pre-COVID-only	One-year-on-only
	n	2916	765	173	819				
	All	62.4	16.4	3.7	17.5				
ACE	0	69.7	13.3	2.7	14.3		Ref		
Count	1	62.9	16.0	3.5	17.6		1.21 (0.98–1.50)	1.33 (0.87–2.03)	1.24 (1.01–1.52)
	2–3	52.9	20.5	4.7	21.9		1.71 (1.36–2.15)	2.00 (1.30–3.08)	1.65 (1.33–2.06)
	≥ 4	40.7	25.8	7.5	26.0	***	2.49 (1.90–3.26)	3.57 (2.26–5.64)	2.27 (1.74–2.96)
Trusted	0	44.7	29.5	4.2	21.6		Ref		
Family	1	50.1	24.7	5.3	19.8		0.90 (0.59–1.37)	1.24 (0.52–2.92)	0.93 (0.59–1.48)
Members (n)	≥ 2	64.6	14.8	3.5	17.1	***	0.54 (0.37–0.78)	0.64 (0.29–1.41)	0.71 (0.47–1.07)
Trusted	0	50.3	25.3	3.5	21.0		Ref		
Friends (n)	1	52.3	24.2	2.4	21.0		0.89 (0.62–1.26)	0.55 (0.24–1.30)	0.87 (0.60–1.26)
	≥ 2	64.7	14.6	3.9	16.8	***	0.64 (0.48–0.85)	1.02 (0.56–1.87)	0.70 (0.53–0.94)
Community	No	56.2	20.2	3.8	19.8		Ref		
Help^a^	Yes	64.4	15.2	3.7	16.8	***	0.78 (0.64–0.94)	0.89 (0.61–1.30)	0.78 (0.65–0.94)
Had	No	63.8	16.4	3.4	16.4		Ref		
COVID-19	Yes	56.3	16.4	5.1	22.2	***	1.08 (0.87–1.34)	1.46 (1.01–2.11)	1.35 (1.11–1.64)
Deprivation	(least) 5	70.7	11.3	3.1	14.9		Ref		
Quintile	4	66.7	12.9	4.8	15.6		1.22 (0.91–1.64)	1.65 (1.01–1.70)	1.10 (0.84–1.43)
	3	60.7	18.8	4.0	16.5		1.87 (1.41–2.48)	1.43 (0.85–2.40)	1.19 (0.91–1.56)
	2	58.9	19.0	3.8	18.3		1.83 (1.38–2.42)	1.29 (0.76–2.18)	1.30 (1.00–1.67)
	(most) 1	56.2	19.5	3.0	21.3	***	1.98 (1.51–2.59)	1.04 (0.61–1.76)	1.61 (1.25–2.06)
Age	18–29	49.9	18.8	6.3	25.1		Ref		
(years)	30–39	53.3	18.4	6.2	22.0		1.00 (0.69–1.45)	1.00 (0.57–1.77)	0.84 (0.60–1.18)
	40–49	59.0	17.8	3.6	19.6		1.03 (0.73–1.46)	0.60 (0.33–1.07)	0.77 (0.56–1.05)
	50–59	61.6	15.9	3.8	18.7		0.90 (0.65–1.26)	0.62 (0.36–1.08)	0.72 (0.53–0.97)
	69–69	64.6	17.0	2.6	15.8		0.98 (0.69–1.38)	0.45 (0.24–0.83)	0.63 (0.46–0.87)
	70 +	72.5	13.5	2.5	11.5	***	0.76 (0.54–1.08)	0.45 (0.25–0.83)	0.44 (0.32–0.62)
Sex	Male	68.7	14.9	2.6	13.8		Ref		
	Female	58.6	17.3	4.4	19.8	***	1.41 (1.18–1.68)	1.96 (1.38–2.79)	1.67 (1.40–1.98)
Ethnicity	White	62.5	16.4	3.6	17.4		Ref		
	Other	61.1	15.2	4.8	18.9	ns	0.77 (0.53–1.13)	1.34 (0.71–2.51)	0.91 (0.64–1.29)
Survey	Phone	64.3	14.8	3.5	17.4		Ref		
Method	Online	52.0	24.8	4.8	18.3	***	1.65 (1.32–2.05)	1.38 (0.91–2.10)	1.03 (0.81–1.30)
Study	England	64.7	16.0	3.0	16.3		Ref		
Location	Wales	61.0	16.6	4.1	18.3	*	1.09 (0.91–1.31)	1.35 (0.95–1.92)	1.25 (1.05–1.49)

*ACE* Adverse childhood experience, *Ref* Reference category

*ns* Not significant

^a^Know where to get help in the community

^*^P < 0.05

^***^P < 0.001

### Patient and public involvement

The study did not involve patients. Study findings are being made publicly available to participants and the general public through the production of study reports and open access journal articles. The study webpages provided contact details for the research team if any individual wished to directly request publications.

## Results

Almost two thirds (62.2%) of participants were female, 64.3% were aged 50 or over and 94.2% were of white ethnicity, with proportions in each deprivation quintile ranging from 17.6% (quintile 3) to 25.0% (quintile 1). Overall, the study sample was a relatively good match across deprivation quintiles to England and Wales population. However, it included a higher proportion of females and individuals of white ethnicity and lower proportions of individuals under 40 years of age (Additional file 1: Table A2). Approximately half of all participants reported 0 ACEs (50.8%), with 22.2% reporting 1 ACE type, 16.7% 2–3 ACE types and 10.3% ≥ 4 ACE types. One in five (19.0%) reported having had COVID-19.

Membership of a poorer mental health category was identified in 25.7% of the sample across time periods. Of these, 6.7% were categorised as *always* poorer mental health (both pre-pandemic and one year on). An additional 15.3% of individuals moved into the category of poorer mental health during the one-year pandemic period (i.e. *one-year-on-only*). A smaller proportion of individuals (3.7%) were categorised as poorer mental health *pre-COVID-only* (Table 1). Similarly, poorer physical health was identified for 32.0% of individuals across any time period. Of these, 10.5% categorised as *always* poorer physical health. An additional 16.4% of individuals moved into the category of poorer physical health during the one-year pandemic period (Table 2). Around five percent were identified as having poorer physical health *pre-COVID-only* (Table 2). Finally, poorer sleep was identified in 37.6% of respondents across any of the time periods. Of these, 16.4% classified as *always* poorer sleepers. An additional 17.5% of individuals moved into the category of poorer sleep during the one-year pandemic period (Table 3). Just under four percent were categorised as poorer sleepers *pre-COVID-only* (Table 3). Further analyses are reported in sections for each outcome variable.

### Mental health

**Fig. 1:**
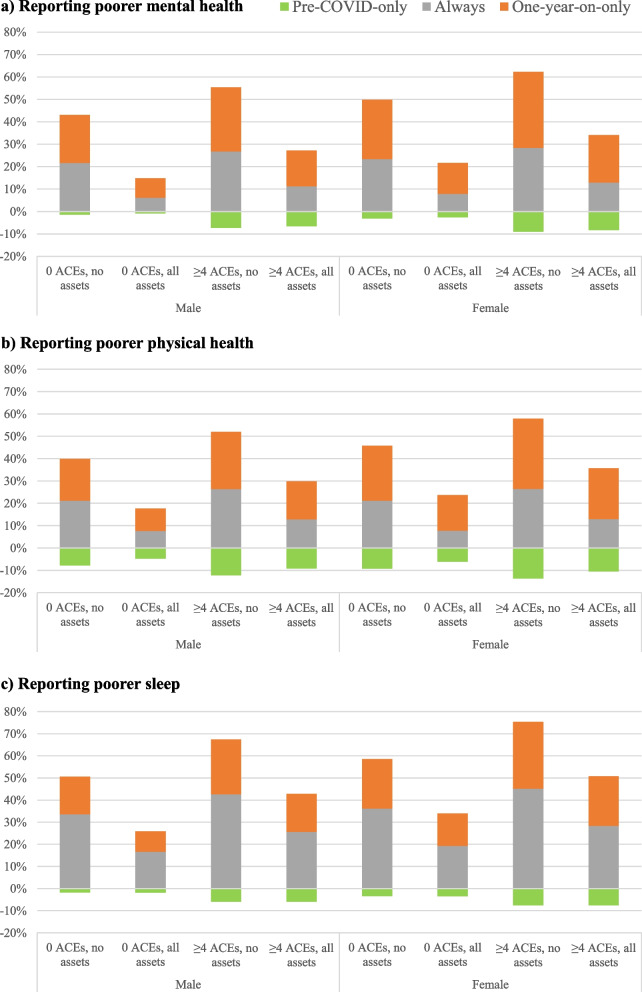
Modelled estimates of proportions with different poor health outcomes one year into the pandemic. Footnote: Stacked bars show percentage membership of poorer well-being category for each time period; *one-year-on-only*, *pre-COVID-only* and *always* (both periods). Percentage movement out of poorer categories from pre-COVID to one-year-on is represented below the x axis. *Pre-COVID-only* represents the proportion of individuals that moved out of poorer outcome categories during the study period. Models estimated are calculated for mid-deprivation (quintile 3), mid-age (40–49 years), white ethnicity and not having had COVID-19. ACE = adverse childhood experiences; no assets = no trusted family members; no trusted friends; do not know where to get help in the local community; all assets =  ≥ 2 trusted family members; ≥ 2 trusted friends; know where to get help in the local community

Bivariate relationships between mental health category and ACEs, social assets and demographics are shown in Table 1. The proportion of individuals in the *never* poorer mental health category reduced with higher ACE counts, fewer trusted family members, fewer trusted friends, and not knowing where to access community help (Table 1). Using MLR (Table 1), ACE counts of ≥ 2 (vs. 0) were associated with membership of all poorer mental health categories (vs. *never* poorer mental health), with odds of poorer mental health *always* or *one-year-on-only* doubling in individuals with ≥ 4 ACEs and odds of poorer mental health *pre-COVID-only* increasing four times. Effect sizes (Cohen’s d, ≥ 4 vs. 0 ACEs) for poorer mental health *always*, *one-year-on-only* and *pre-COVID-only* were 0.47 (small), 0.41 (small) and 0.81 (large) respectively. Having ≥ 2 trusted family members (vs. 0) reduced odds of each poorer mental health category, with odds of poorer mental health *one-year-on-only* also reduced with one trusted family member. Having ≥ 2 trusted friends (vs. 0) and knowing where to access community help reduced odds of *always* poorer mental health only. Demographically, *always* poorer mental health was also associated with greater deprivation, being younger, female and white, as well as completing the survey online (Table 1). Younger age and being female were the only other factors significantly related to poorer mental health *pre-COVID-only*. Younger age, being female, having had COVID-19, residence in the most deprived (vs. least deprived) quintile and completing the survey online were also associated with poorer mental health *one-year-on-only* (Table 1). MLR models were used to estimate demographically adjusted percentage changes in mental health category membership over the pandemic period for specific socio-demographics (see Methods). Thus, for women (mid-deprivation quintile, aged 40–49 years) an estimated 13.8% of individuals with no ACEs and high supporting assets (friends, family, community help knowledge) moved into poorer mental health during the pandemic (i.e. poor mental health *one-year-on-only)*, rising to 33.8% of those with ≥ 4 and low access to support (Fig. 1a). Moreover, 2.5% of women with no ACEs and high supporting assets moved out of poorer mental health over the year (i.e. poor mental health *pre-COVID-only*) rising to 9.0% of those with ≥ 4 ACEs and low access to support (Fig. 1a).

### Physical health

In bivariate analyses, the proportion of individuals in the *never* poorer physical health category reduced with ACE count, fewer trusted family members, fewer trusted friends and not knowing where to access community help (Table 2). In MLR, odds of poorer physical health *one-year-on-only* were significantly elevated with any ACE exposure (Table 2)*.* Odds of *always* poorer physical health were increased in those with ≥ 2 ACEs and of poorer physical health *pre-COVID-only* in those with ≥ 4 ACEs (vs. 0 ACEs; Table 2). Effect sizes (Cohen’s d, ≥ 4 vs. 0 ACEs) for poorer physical health *always*, *one-year-on-only* and *pre-COVID-only* were 0.40 (small), 0.38 (small) and 0.51 (medium) respectively. Having ≥ 2 (vs. 0) trusted family members was associated with reduced odds of all poorer physical health categories while having ≥ 2 trusted friends (vs. 0) and knowing where to access community help reduced odds of *always* poor physical health. *Always* poorer physical health (vs. *never*) was also associated with greater deprivation, white ethnicity, online participation and being resident in Wales. Poorer physical health *pre-COVID-only* was associated with younger age, being female, online participation, and inconsistently associated with deprivation, with greatest risk in the second most affluent quintile. Poorer physical health *one-year-on-only* was associated with the highest deprivation quintile (vs. the lowest), younger ages, having had COVID-19 and being female. Using the MLR models an estimated 10.1% of males (deprivation quintile 3, age 40–49 years) with no ACEs and high supporting assets (friends, family, community help) moved to poorer physical health during the pandemic (i.e. *one-year-on-only* category) rising to 25.6% of those with ≥ 4 ACEs and low access to support (Fig. 1b). In the same demographic, 4.8% moved out of poorer physical health over the year (i.e. *pre-COVID-only* category) rising to 12.2% of those with ≥ 4 and low access to support (Fig. 1b).

### Poorer sleep

The proportion of individuals in the *never* poorer sleep category reduced with higher ACE counts, fewer trusted family members, fewer trusted friends and not knowing where to access community help (Table 3). Using MLR, ACE counts of ≥ 2 (vs. 0) were associated with membership of all poorer sleep categories (vs. *never* poorer sleep), with poorer sleep *one-year-on-only* also elevated with one ACE. Odds of poorer sleep *pre-COVID-only* more than tripled in individuals with ≥ 4 ACEs and odds of poorer sleep *always* or *one-year-on-only* more than doubled (Table 3). Effect sizes (Cohen’s d, ≥ 4 vs. 0 ACEs) for poorer sleep *always*, *one-year-on-only* and *pre-COVID-only* were 0.50 (medium), 0.45 (small) and 0.70 (medium) respectively. Having ≥ 2 trusted family members (vs. 0), ≥ 2 trusted friends (vs. 0) and knowing where to access community help reduced odds of always poorer sleep. Having no trusted friends and not knowing where to access community help were also associated with poorer sleep *one-year-on-only*, although no social asset measures were associated with *pre-COVID-only* poorer sleep. Demographically, *always* poorer sleep was significantly associated with higher levels of deprivation, being female and online survey completion (Table 3). Younger age, being female and having had COVID were the only other factors significantly related to poorer sleep *pre-COVID-only*, while high deprivation, younger age, being female, having had COVID-19 and being from Wales were also associated with poorer sleep *one-year-on-only*. Using MLR models to estimate demographically adjusted percentage changes in sleep category membership over the pandemic period, for women (mid-deprivation quintile, aged 40–49 years) an estimated 14.6% with no ACEs and high supporting assets (friends, family, community help) had moved into poorer sleep (*one-year-on-only* category) rising to 30.2% of those with ≥ 4 and low supporting assets (Fig. 1c). Further, of the same demographic, 3.5% moved out of poorer sleep (*pre-COVID-only* category) over the year, rising to 7.6% of those with ≥ 4 ACEs and low access to support (Fig. 1c).

## Discussion

Consistent with many studies, results here identify those with a history of higher childhood adversity (ACEs) as being more likely to have entered the pandemic with poorer mental health, physical health and sleep quality. Thus, for those with ≥ 4 ACEs, 23.2% were categorised as having poorer mental health pre*-*COVID (categories of *always* or *pre-COVID-only* combined), 27.3% as having poorer physical health and 33.3% as having poorer sleep compared to 7.3%, 12.8% and 16.0% respectively in those with no ACEs (Tables 1, 2 and 3).

As with other studies [2], nearly half of all adults surveyed had experienced at least one ACE and one in 10 had suffered ≥ 4 ACEs. Consequently, ACEs represent a marker of existing vulnerabilities which may be overlooked in crisis situations such as pandemics, but which affect large proportions of populations.

Even amongst those with ACEs but, as yet, better mental and physical health, our results suggest ACEs are associated with movement towards a poorer health outcome during the pandemic. Those with ≥ 4 ACEs were around twice as likely to have moved into the poorer mental health, physical health and sleep categories over the pandemic although effects sizes for movement towards poorer outcomes over the pandemic (≥ 4 vs 0 ACEs) across all outcomes were relatively small. For physical health and sleep, increased likelihood of movement into poorer categories was apparent with just one ACE, with effects significant on mental health from ≥ 2 ACEs (Tables 1, 2 and 3). Results here are consistent with a history of ACEs being associated with lower resilience in crisis [24, 27]. Consequently, individuals’ abilities to adapt to change and accommodate reduced levels of social and professional support in the pandemic may contribute to more detrimental impacts on health and well-being (Tables 1, 2 and 3).

Studies on population health during the pandemic have focused primarily on factors associated with increasing harms and less on where health and well-being may have improved [28, 29]. Individuals with higher ACEs may be less likely to feel well adapted to work and social environments even in non-pandemic times [30, 31] and consequently changes, for some, may not necessarily be negative. Here, individuals with ≥ 4 ACEs were also more likely to move out of poorer mental health (4.36 times), physical health (2.54 times), and sleep categories (3.57 times) during the pandemic. Moreover, whilst differences were significant for all outcomes with ≥ 4 ACEs they were also significant for mental health and sleep at ≥ 2 ACEs (vs. no ACEs, Tables 1, 2 and 3). Effect sizes (≥ 4 vs. 0 ACEs) for movement out of poorer outcomes categories during the pandemic were greater than those for movement in with, for instance, a large effect size associated with movement out of the poorer mental health category. The pandemic may have provided changes in working (e.g. from home, flexible times, etc.), social pressures (in or out of work) or other stressors that benefited a sub set of individuals [32], such as those with ACEs. Consequently, such vulnerable individuals may also benefit from different post-pandemic models of work and socialising with further studies required to identify how these models might best be realised.

Results here also explored which social assets were associated with better health outcomes during the pandemic. Having higher numbers of trusted family members or friends (≥ 2 vs. 0) was strongly associated with lower likelihood of *always* reporting poorer mental health, physical health and sleep (Tables 1, 2 and 3). More trusted family members was also associated with more than halving the likelihood of developing poorer mental and physical health over the pandemic but had no impact on sleep quality. However, higher number of trusted friends was related to improvement in sleep quality (Tables 1, 2 and 3). Why results varied from family to friends between outcomes requires further examination. Together though, these findings support other studies [18, 33, 34] that suggest having multiple trusted individuals may contribute to better physical and mental health outcomes including in crisis situations. Higher number of trusted family members was also associated with reduced likelihood of moving out of poorer mental and physical health categories during the pandemic (Tables 1 and 2) which is consistent with family support being protective against poorer health pre-COVID. Whilst this study did not specifically measure communications with friends and family during the pandemic, results suggest a vital role for retaining connectedness during pandemics or other crises.

Finally, we found knowing where to get help in the local community was associated with lower likelihood of *always* (i.e. both before the pandemic and one year one) reporting poorer mental, physical and sleep outcomes. Otherwise, community support was only associated with lower risks of moving into poorer sleep during the pandemic (Tables 1, 2 and 3). However, we did not measure respondents’ history of seeking help from health care or other services locally either prior to or during the pandemic. Such contact, especially during the pandemic, may be related to knowing where to get help in the community and could have contributed to changes in well-being outcomes. A history of ACEs, few or no trusted friends and family, and not knowing where to access help in the community each contribute only parts of the risk for poorer outcomes across the pandemic. However, individuals who experience all of these appear to have had dramatically different health trajectories. For instance, 28.7% of men with ≥ 4 ACEs, no trusted friends or family members and no knowledge of where to access community help moved into the poorer mental health category during the study period compared to 8.6% of those with no ACEs and all these assets (Fig. 1a, modelled estimate for men, deprivation quintile 3, aged 40–49, white, who did not report having had COVID-19). Similar differences depending on ACEs and social assets are apparent across all outcomes (Fig. 1a-c).

Whilst not the focus of this study, examination of other socio-demographic and pandemic experiences identified some associations of interest. Consistent with other studies, reporting having had COVID-19 was associated with increased risks of developing poorer mental health, physical health and sleep [35, 36]. This was also the case for being resident in areas of high deprivation and being female ([37]; Tables 1, 2 and 3). Also, as reported elsewhere [16, 36], younger ages showed a strong relationship with moving to poorer mental health during the pandemic (Table 1). However, younger age was also associated with movement into poorer physical health during the pandemic as well as *always* having poorer physical health (Table 2, see limitations).

### Limitations

Undertaking a survey during a pandemic created challenges relating to access to respondents, urgency in developing and delivering data collection tools covering a wide range of topics, and the need for widely understandable approaches to presentation and communication of findings to inform action. Such issues required pragmatic epidemiological approaches which also resulted in some limitations. Questions on health outcomes were developed specifically for this survey and used a single item measure for each outcome. Piloting identified no issues with understanding and response rates. However, more work on validation of these measures is required in future studies to understand their limitations and how they may be refined for use in further studies undertaken in restrictive circumstances. Further, health measures were self-assessed, thus respondents are likely to have judged their health and sleep quality relative to personal expectations rather than against any objective scale. There is some suggestion in our data that such expectations may have been higher in young people and consequently scores for current health and sleep lower (Tables 1, 2 and 3). Measures of pre-COVID mental health, physical health, and sleep relied on retrospective self-assessment which could have introduced recall errors and biases. For instance, a study comparing prospective and retrospective measures of anxiety and depression symptoms in individuals with pre-existing mental health conditions found correlation between retrospective and prospective measures, yet that retrospective measures tended to underestimate prior symptom severity [38]. Here, we could not measure whether different groups (e.g. those with or without ACEs) may have been affected differently by recall bias and consequently if recall may have impacted findings.

ACEs were also self-reported retrospectively. Although surveying adults about their history of ACE exposure is a well-established methodology, recall bias remains a potential issue. Studies suggest both retrospective and prospective ACE measures show similar associations with health and well-being outcomes [39, 40]. However, retrospective measures of ACEs may overestimate relationships with subjectively measured poorer health outcomes [40]. We were not able to collect objective independently assessed health measures during the pandemic and surveying adults necessitated retrospective measurement of ACEs. Further work is required on the accuracy and utility of ACE data collected both prospectively and retrospectively and on the robustness of relationships between these measures and both subjective and objectively collected outcome measures.

A key aspect of this study was to identify factors associated with having or avoiding a poorer level for each well-being outcome. The study did not aim to identify individuals with clinical needs or relate respondents’ health and well-being ratings to a particular diagnosis. Rather, as a broader population study it categorised self-reported measures into higher or lower categories for each outcome and allowed analysis of outcome membership pre-pandemic and one year on as well as any transition between categories over this time. Consequently, we used a binary measure splitting respondents according to whether they self-rated in the lower (0–5; poorer for all outcomes) or higher (6–10) halves of the scales. No post hoc attempt to create different category boundaries for outcomes was undertaken. Whilst those rating themselves in the lower half of the scale are more likely to report health and well-being vulnerabilities, some individuals with well-being issues will not have been captured in the poorer category. Further work is required to assess how such limited measures compare to more extensive validated scales and whether binary upper and lower scale membership is an appropriate method for their categorisation. Overall, however our findings on poorer levels of mental and physical health in those with higher ACEs are consistent with other studies [2]. Other potential sources of variation (e.g. substance use, education level) were not included but may also contribute to well-being and changes in well-being through the pandemic. Our study identifies associations and not necessarily causal relationships between ACEs and poorer outcomes across mental health, physical health and sleep measures. Other studies have now identified biological changes associated with exposure to ACEs which provide stronger evidence for a causal role for ACE in poorer health and well-being [41]. In this study however, we cannot directly rule out a role for other factors, either genetic or environmental, which may correlate with both ACEs and poorer health outcomes and consequently contribute to observed associations between the two. Finally, although this study used a substantive sample (*n* = 4,673), compliance was 33.3% through telephone contacts and not measurable through the online component. Less than 5% of individuals were removed due to incomplete data and whilst we adopted a complete case analysis approach, we are not able to examine any potential bias introduced into the data through sample self-selection or incomplete responses.

## Conclusions

Nearly everyone has faced substantive changes and challenges to their lives as a result of the pandemic. Across the world, populations have experienced increased isolation coupled with reduced access to physical activities, social and professional support and shaken confidence in their financial futures [42–44]. Recently, a variety of systems across health, education and criminal justice have begun to adopt trauma informed approaches to the delivery of their services and support. Such approaches ensure strategies and staff recognise that individuals with a history of trauma, such as ACEs, may require additional or different support when facing crises or other life course challenges [45]. Our results suggest that ACEs may leave people’s general mental and physical health at greater risk in pandemic situations and childhood adversity should be considered when assessing vulnerability in future pandemics. Our results also support the importance of friends and family in crises and the additional health risks those without such connections face. Although we are not aware of comparable data from other public health crises, such vulnerabilities linked to early adversity and lack of trusted peers may be common to multiple settings and requires further examination. Our findings revealed a smaller subset of individuals with high ACEs whose health outcomes improved during the pandemic. Thus, whilst aspects of home working, virtual communication and greater isolation may be harmful to some groups, it may suit others better. Current debate over post COVID norms must take into account the heterogeneity in how proposed changes and new norms will affect health in different populations. Creating the right environments for people already struggling with a history of adversity may help improve their health and happiness. Creating the wrong ones may exacerbate physical and mental health issues and encourage a cycle of ACEs passing from one generation to another.

### Supplementary Information

**Additional file 1: Table A1.** Questions and qualifying responses for independent variables.** Table A2.** Participant characteristics and variable distributions with demographic comparison to England and Wales population.

## Data Availability

The dataset analysed in the current study is available from the corresponding author on reasonable request.

## Abbreviations

ACE: Adverse childhood experience
IMD: Index of multiple deprivation
MLR: Multinomial logistic regression
MRC: Market research company
NS: Not significant
Ref: Reference category
