# Supplementary material for: Measuring changes in adult health and well-being during the COVID-19 pandemic and their relationship with adverse childhood experiences and current social assets: a cross-sectional survey
Source: BMC Public Health. 2023 Aug 24;23:1618. doi: 10.1186/s12889-023-16549-z (PMC10463476; doi:10.1186/s12889-023-16549-z)
Supplement: Supplementary file 1 — Additional file 1: Table A1. Questions and qualifying responses for independent variables. Table A2. Participant characteristics and variable distributions with demographic comparison to England and Wales population. [file 12889_2023_16549_MOESM1_ESM.docx]

**Table A1. Questions and qualifying responses for independent variables**

|  | **Question (response options)** | **Qualifying response** |
| --- | --- | --- |
| **ACEs** | All ACE questions were preceded by the statement “While you were growing up, before the age of 18...” | |
| Physical abuse | How often did a parent or adult in your home ever hit, beat, kick, or physically hurt you in any way? This does not include gentle smacking for punishment. (never; once; more than once; prefer not to say) | Once or more than once |
| Verbal abuse | How often did a parent or adult in your home ever swear at you, insult you, or put you down? (never; once; more than once; prefer not to say) | More than once |
| Sexual abuse | Did an adult or someone at least five years older than you sexually abuse you by touching you or making you undertake any sexual activity with them? (yes; no; prefer not to say) | Yes |
| Parental separation | Were your parents ever separated or divorced? (yes; no; prefer not to say) | Yes |
| Domestic violence | How often did your parents or adults in your home ever slap, hit, kick, punch, or beat each other up? (never; once; more than once; prefer not to say) | Once or more than once |
| Mental illness | Did you live with anyone who was depressed, mentally ill or suicidal? (yes; no; prefer not to say) | Yes |
| Alcohol abuse | Did you live with anyone who was a problem drinker or alcoholic? (yes; no; prefer not to say) | Yes |
| Drug abuse | Did you live with anyone who used illegal street drugs or abused prescription medications? (yes; no; prefer not to say) | Yes |
| Incarceration | Did you live with anyone who served time or was sentenced to serve time in a prison or young offenders' institution? (yes; no; prefer not to say) | Yes |
| **Social assets** | |  |
| Trusted family members | Roughly how many close family members do you have that you trust (none; 1; 2-5; 6-10; 11-20; 21-30; over 30) | Categorised 0, 1 2+ |
| Trusted friends | Outside of your family, roughly how many close friends do you have that you can trust (none; 1; 2-5; 6-10; 11-20; 21-30; over 30) | Categorised 0, 1 2+ |
| Community help | Currently, do you know where to get help in your community (yes; no) | Yes |
| **COVID-19 status** |  |  |
| Had COVID-19 | Do you think you have had coronavirus? (or currently have it) (yes; no; don’t know) | Yes |

ACE = Adverse childhood experience.

**Table A2: Participant characteristics and variable distributions with demographic comparison to England and Wales population**

|  |  | n | % | England & Wales population (%) |
| --- | --- | --- | --- | --- |
| Total |  | 4673 | 100.0 |  |
| ACE count | 0 | 2372 | 50.8 | NA |
|  | 1 | 1038 | 22.2 |  |
|  | 2-3 | 782 | 16.7 |  |
|  | ≥4 | 481 | 10.3 |  |
| Trusted family members | 0 | 190 | 4.1 | NA |
|  | 1 | 449 | 9.6 |  |
|  | ≥2 | 4034 | 86.3 |  |
| Trusted friends | 0 | 400 | 8.6 | NA |
|  | 1 | 409 | 8.8 |  |
|  | ≥2 | 3864 | 82.7 |  |
| Community help^a^ | No | 1118 | 23.9 | NA |
|  | Yes | 3555 | 76.1 |  |
| Had COVID-19 | No | 3783 | 81.0 | NA |
|  | Yes | 890 | 19.0 |  |
| Deprivation quintile | (least) 5 | 931 | 19.9 | 18.9 |
|  | 4 | 910 | 19.5 | 20.5 |
|  | 3 | 824 | 17.6 | 20.7 |
|  | 2 | 841 | 18.0 | 20.3 |
|  | (most) 1 | 1167 | 25.0 | 19.7 |
| Age (years) | 18-29 | 399 | 8.5 | 18.7 |
|  | 30-39 | 499 | 10.7 | 17.2 |
|  | 40-49 | 771 | 16.5 | 15.9 |
|  | 50-59 | 1022 | 21.9 | 17.3 |
|  | 69-69 | 896 | 19.2 | 13.6 |
|  | 70+ | 1086 | 23.2 | 17.3 |
| Sex | Male | 1767 | 37.8 | 48.4 |
|  | Female | 2906 | 62.2 | 51.6 |
| Ethnicity | White | 4403 | 94.2 | 83.9 |
|  | Other | 270 | 5.8 | 16.1 |
| Survey method | Phone | 3948 | 84.5 | NA |
|  | Online | 725 | 15.5 |  |
| Study location | Bolton | 1825 | 39.1 | NA |
|  | Wales | 2848 | 60.9 |  |

ACE = Adverse childhood experience. ^a^Know where to get help in the community. NA = not applicable.

National demographic data sources: Office for National Statistics

Deprivation quintile: England: Population by Index of Multiple Deprivation (IMD), England, 2001 to 2019. <https://www.ons.gov.uk/peoplepopulationandcommunity/populationandmigration/populationestimates/adhocs/12386populationbyindexofmultipledeprivationimdengland2001to2019> Wales: calculated from Lower layer Super Output Area population estimates, <https://www.ons.gov.uk/peoplepopulationandcommunity/populationandmigration/populationestimates/datasets/lowersuperoutputareamidyearpopulationestimates>

Age and Gender: Mid-Year Population Estimates, UK, June 2021. <https://www.ons.gov.uk/peoplepopulationandcommunity/populationandmigration/populationestimates/datasets/populationestimatesforukenglandandwalesscotlandandnorthernireland>

Ethnicity: Ethnic group, England and Wales: Census 2021. <https://www.ons.gov.uk/peoplepopulationandcommunity/culturalidentity/ethnicity/bulletins/ethnicgroupenglandandwales/census2021>
